# The worst is yet to come: probable sporadic Creutzfeldt–Jakob disease in a well-controlled HIV patient

**DOI:** 10.1080/19336896.2019.1648985

**Published:** 2019-08-12

**Authors:** Euripedes Gomes De Carvalho Neto, Matheus Ferreira Gomes, Marina De Oliveira, Maryuris Isabel Niño Guete, Iuri Pereira Santos, Mateus Damiani Monteiro, Fernando Gustavo Stelzer, Fernando Kowacs, Liselotte Menke Barea

**Affiliations:** a Department of Neurology, Universidade Federal de Ciências da Saúde de Porto Alegre, Irmandade Santa Casa de Misericórdia de Porto Alegre, Porto Alegre, Brazil; b Irmandade da Santa Casa de Misericórdia de Porto Alegre, Porto Alegre, Brazil

**Keywords:** EEG, HIV, MRI, prion, prion disease

## Abstract

We describe a case of probable sporadic Creutzfeldt–Jakob disease in the setting of well-controlled HIV and discuss whether exist, in fact, HIV-related factors that may predispose to the development of prion disease. To the best of our knowledge, this is the third report of this association.

## Introduction

Creutzfeldt–Jakob disease (CJD) is a rare, fatal, rapidly progressive neurodegenerative human disorder occurring in sporadic, genetic, and acquired forms. Sporadic CJD (sCJD), by far the most common form, is typically characterized by a rapidly progressive clinical course and widespread brain deposition of abnormal prion protein (PrPTSE) aggregates leading to spongiform change, gliosis, and neuronal loss [1].

We describe a case of this rare disease in the setting of well-controlled HIV and discuss whether exist, in fact, HIV-related factors that may predispose to the development of prion disease. To the best of our knowledge, this is the third report of this association [2,3]. Another case of CJD had been recently described in a patient with a recent diagnosis of AIDS [4].

## Case report

A 52-years-old Caucasian male with well-controlled HIV came to the emergency department presenting progressive imbalance, motor and cognitive deterioration and hypersomnia. Before being referred, he was evaluated by a psychiatrist who prescribed neuroleptics. On admission, he was in a state of mutism and did not obey to commands, although there were no vital signs abnormalities, meningism or focal motor deficit. His motor examination showed global deep tendon hyperreflexia and increased tone in all extremities with intermittent myoclonus.

Recent CD4 count was 495 µL^−1^ and HIV-1 RNA was undetectable. Initial laboratory work-up was unremarkable. Serum cryptococcal antigen, Toxoplasma IgM, Cytomegalovirus IgM, Herpes I and II and fluorescent treponemal antibody-absorption test (FTA-ABS) for syphilis were negative. Routine CSF analysis was normal: total protein 30 mg/dL, glucose 59 mg/dL (serum glucose, 80 mg/dL), 5 red blood cells, and 3 mononucleated cells. Opening pressure was 160 mmH_2_O. CSF polymerase chain reaction for herpes simplex virus, cytomegalovirus, tuberculosis, and JCV were negative, as well as VDRL, cryptococcal antigen and bacterial and fungal cultures.

**Figure 1.**
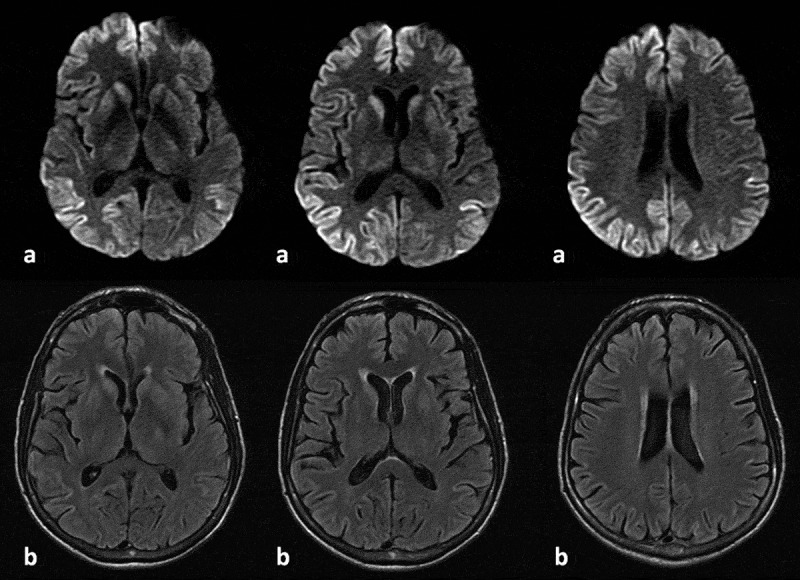
(a) Axial diffusion-weighted image (DWI) sequences show diffusion restriction of water molecules along the bilateral caudate and gyriform cortical-restricted diffusion in the bilateral cerebral hemispheres. (b) Axial fluid-attenuated inversion-recovery (FLAIR) images sequences show discrete areas of hypersignal in bilaterally caudate nuclei, as well as in the cortical regions.

**Figure 2.**
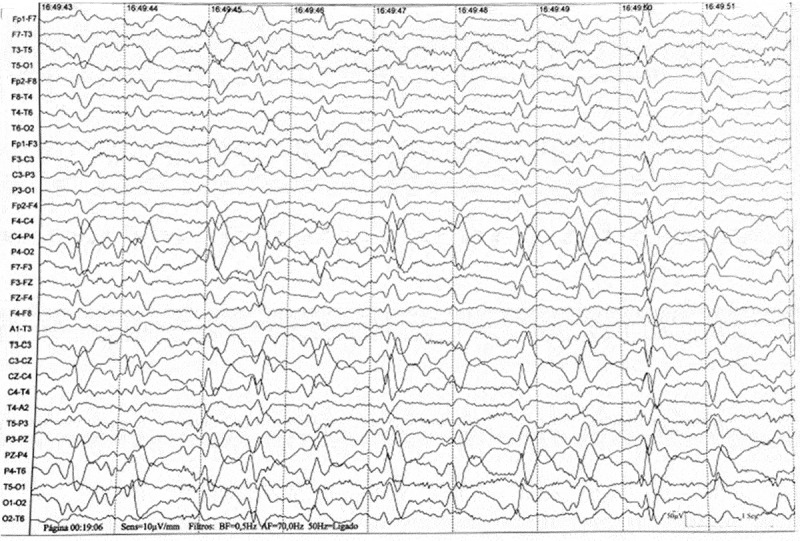
Electroencephalography (EEG) showing periodic sharp wave complexes (PSWC) every 1 s. There is also severe disorganization of the base rhythms, which are composed of irregular and diffuse slow activities.

Diffusion-weighted brain MRI showed cortical gyriform restriction on both hemispheres and on both caudate nuclei (Figure 1). EEG showed 1 Hz triphasic periodic sharp wave complexes (PSWC) (Figure 2) and CSF 14–3-3 protein level was elevated (77,849 AU/mL; reference value: <20,000 AU/mL). The combination of rapidly progressive mental state and motor deterioration and typical CSF, radiographic, and electroencephalographic features pointed to probable CJD according to CDC’s criteria[5]. The patient died after 2 months of hospitalization from respiratory sepsis.

## Discussion

Many diagnostic hypotheses should be considered in the context of an HIV-patient presenting with cognitive decline, e.g. HIV-associated encephalopathy, central nervous system opportunistic infections, and immune reconstitution syndrome. Although cognitive impairment can also be observed in patients with well-controlled HIV infection, rapidly progressive dementia is rare [4].

HIV-associated neurocognitive decline is characterized by subcortical dysfunction, attention, concentration and psychomotor impairment and mood disorder [6]. In this patient, rapid progression of the symptoms and the long period of well-controlled HIV infection made this diagnosis less likely. CNS opportunistic infections, on the other hand, were ruled out by neuroimage and CSF analysis.

Brain MRI also ruled out structural abnormalities such as tumour or stroke, but DWI and FLAIR sequences revealed bilateral diffusion restriction and hyperintensity in the caudate nuclei and cerebral cortex, the latter affected in a ribbon pattern (Figure 1). Cortical and basal ganglia involvement, with insula and cingulate cortices hyperintensity at DWI, in addition to involvement of the superior frontal gyri and cortical areas near the midline are the most common MRI pattern in sCJD [7].

Electroencephalography findings may provide additional supportive evidence for CJD. The most distinctive pattern is characterized by periodic sharp wave complexes that occur in the middle and late stages of the disease [8]. Our patient exhibited these classical EEG findings along with elevated CSF 14-3-3 protein which, although not specific, reinforces the clinical hypothesis of sCJD [5,9,10].

A well-controlled HIV-patient that developed sCJD was the subject of a recent report by Babi and colleagues [2]. The authors theorized that HIV infection could have predisposed to prion disease, based on the fact that both pathogens, despite being essentially different, depend on interfering with cholesterol cell efflux pathway for replication and infection[11]. HIV exploits the regulation of ATP-binding cassette transporter type 1 (ABCA1) functionality by displacing it from neuronal membrane lipid rafts to the intracellular compartments, and this effect on ABCA1 and lipid rafts was also observed in a prionic disease animal model [11,12].

## Conclusion

As far as we know, this is the third report of sCJD affecting a chronic well-controlled HIV patient. Although shared cellular mechanisms had been demonstrated [2,12], no causal link between HIV and spontaneous CJD has yet been firmly established.
